# Assessment of the potential ecological and human health risks of heavy metals in Ahvaz oil field, Iran

**DOI:** 10.1371/journal.pone.0242703

**Published:** 2020-11-24

**Authors:** Mahmood Reza Ghorbani, Navid Ghanavati, Timoor Babaenejad, Ahad Nazarpour, Khoshnaz Payandeh

**Affiliations:** 1 Department of Soil Science, Ahvaz Branch, Islamic Azad University, Ahvaz, Iran; 2 Department of Geology, Ahvaz Branch, Islamic Azad University, Ahvaz, Iran; Zhongnan University of Economics and Law, CHINA

## Abstract

The potential hazard to human health from exposure to heavy metals in surface soil was assessed using 66 soil samples collected from Ahvaz oil field. To this end, the contents of heavy metals were measured by the inductively coupled plasma spectroscopy (ICP-OES). Mean levels of As, Cd, Co, Cr, Cu, Ni, Pb, V, and Zn were 5.9, 0.4, 7.1, 36.5, 41.2, 39.8, 67.4, 31.5, and 77.6 mg/kg, respectively. Contents of all studied heavy metals, with the exception of Co, Cr, and V, were several times higher than that of baselines. Correlation coefficients and principal component analysis (PCA) identified two main groups as sources of heavy metals in the surface soil of Ahvaz oil-field. Metals such as Co, Cr, and V were observed to originate from natural sources and As, Cd, Cu, Ni, Pb, and Zn originated from anthropogenic sources such as petroleum leakage and the pollution caused by drilling mud from oil wells. Pb and Zn were of significantly high EF mean enrichment value, and Co, Cu, Cd, and As had high enrichment in surface soil. Pb, Cr, V, Zn, Co, Cu, Ni, and As had a low potential ecological risk (PER) whereas Cd had a moderate PER. The risk of carcinogenic and non-carcinogenic diseases was detected to be higher in children than in adults. The carcinogenic risk (Cr) calculation was more than 1 × 10^-6^ for children and adults. Additionally, the CR of Cr for both children and adults indicated risk under control conditions.

## Introduction

Over the past two decades, special attention has been paid to the ramifications of environmental pollution due to the growing needs of the population, the development of land and mineral resources, and the creation of a wide range of chemical pollutants including heavy metals [1,2]. As a stable complex, soil is in contact with other environmental components such as air and water; therefore, contaminants can spread to the surface and groundwater as well as air, thereby polluting them [3]. The most important soil pollutants are heavy metals, acid precipitation, and organic matters, among which heavy metals have attracted considerable attention owing to their non-degradable, toxic, and carcinogenic properties [4]. In general, heavy metals are present in the evironment via anthropological and natural mechanisms. Indeed, human activity may lead to the buildup of more heavy metals in the soil. All heavy metals are toxic to the soil at concentrations higher than normal levels [5]. The oil industry is one of the key industries in Iran and the rest of the world owing to its critical role in supplying energy and generating raw materials for many other industries. Moreover, a highly significant source of soil heavy metal pollutants exists in this industry. Despite its importance, the oil industry is also considered as one of the polluting industries [6]. Crude oil is a complex combination of organic (polycyclic aromatic hydrocarbons) and inorganic components [7]. A plethora of heavy metal contents are found in crude oil, varying with the type of oil extracted in different regions. Accordingly, the pollution from heavy metals is an environmental issue associated with the terrestrial ecosystems of oil-producing countries [8]. The construction and production of refineries are among the activities that lead to the release of heavy metals into the soil. It is also possible that petroleum hydrocarbons contaminate the soil during oil refining and treatment processes. However, along with these petroleum pollutants, heavy metals such as Ni, V, Pb, Cd, Zn, and Cu are added to the soil [9–11]. The high concentration of such heavy metals has myriad negative effects on human health, mainly through ingestion, inhalation, and dermal contact. Children are reported to absorb more of these metals compared with adults, which disrupts their metabolic behavior [12]. Besides, the excessive consumption of heavy metals by individuals causes acute and chronic toxicities such as damage to the central nervous system, blood, lung, kidney, and liver and death. Nazarpour et al. [4] assessed the amount of contamination and the potential ecological risk (PER) of certain heavy metals in the oil-field surface soil; using the Nemerow integrated pollution index (NIPI), they detected a remarkable amount of pollution in all metals, except for V. Moreover, in a study on the PER of the examined metals, V, Cd, Zn, and Cr were less risky ecologically, and Cu had moderate ecological risk whilePb and Ni showed significant risks. Moreover, Ebrahimi et al. [13] reported that the possible oil leakage from storage tanks, pipe fractures, or sewage canals contaminated the soil around Sarkhoon Gas Refinery in Bandar Abbas (Iran) with oil contaminants. They further showed that soil contamination in this area augmented other pollution index parameters, including total soluble solids, suspended solids, and chemical and biological oxygen demand. Similarly, Fasihi et al. [14] studied the environmental pollution status of petroleum compounds and heavy metals in Tehran, concluding that the area was highly polluted in terms of air and soil. The high concentration of these compounds in the soil was reported to be detrimental to human health [15]. As shown in the findings, the soil Ni and V did not exceed the standard level; however, their relatively high amount in the soil of the research zone was considered as a risk to human health and environment. Accordingly, the environmental importance of this issue necessitates investigating the concentrations of heavy metals in Ahvaz oil field as the largest crude oil field in Iran. Ahvaz oil field (67 km long and about 6 km wide) is Iran's largest oil field located in Khuzestan province, southwest of Iran. It is also Iran’s largest crude oil-field in terms of its crude oil storage capacity and the world's third largest oil field after Ghawar (Saudi Arabia) and Burgan (Kuwait). On average, the crude oil production capacity of Ahvaz oil field is 800,000 barrels per day; its gas production capacity (gas plus oil) is over 13 million cubic meters per day. The field's crude oil storage capacity is estimated at more than 65 billion barrels, from which approximately 37 billion barrels can be extracted on average.

Hence, this study aimed to 1) determine the levels of heavy metals such as Pb, Ni, Zn, Cu, Cd, Cr, As, V and Co in the soil surrounding the drilling rigs and oil installations in Ahvaz oil-field; 2) use statistical analyses such as Pearson's correlation coefficients, principal component analysis (PCA), and cluster analysis (CA) to specify the relationships between different elements used in detecting the natural or anthropogenic sources of such elements and understand how they are transferred in the environment; and 3) evaluate the risks of heavy metals on human health and environment based on environmental indices such as enrichment factor, geoaccumulation index, potential ecological risk, and human health risk.

## Materials and methods

### Sampling

**Fig 1:**
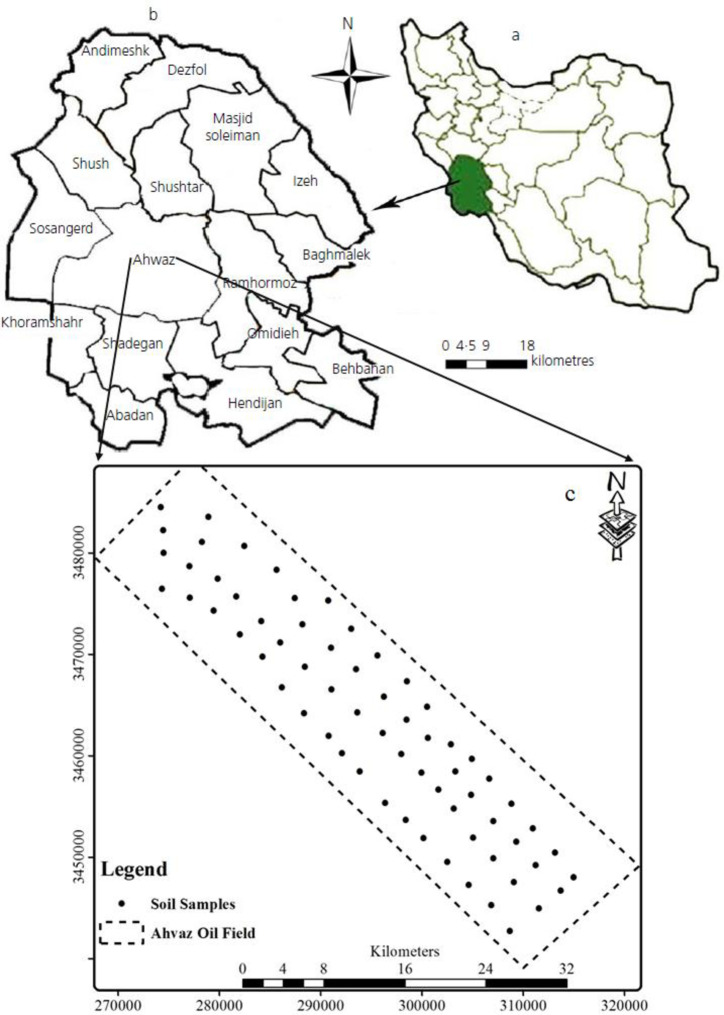
Location map of (a) Iran (b) Khuzestan Province and (C) border of Ahvaz oil field and soil sampling sites.

In this study, environmental indices were assessed through sampling some heavy metals in the surface soil of Ahvaz oil field in June 2018. The distribution map of the sampling points is shown in Fig 1. Using a mixture of random and systematic point sampling distribution in ArcGIS sofware version 10.4, the authors specified the sampling and collection strategies. Afterwards, through modifying the site of each sampling location, the Universal Transverse Mercator (UTM) of each site was applied to GPS to immediately access the sampling location. The points were selected so that the entire study area was covered. Mixed samples (six mixed samples from 5 to 10 m apart) with an approximate weight of 500 grams were further collected. This paper is from the first author’s PhD thesis, who is employed in National Iranian Oil Company (NIOC), and all of the soil samples were collected with coordination process of NIOC license. Ahvaz oil field is located in a wide open area and we have a license from our university and NIOC as permission for soil sample collection. Next, 66 samples were collected from 0-10 cm depth by authors, packed in plastic bags, and transferred to the laboratory. The samples were then air-dried, passed through a 220 mesh (63 μm), and stored in polyethylene bags, and labeling was carried out.

### Extraction and analysis of heavy metals

Heavy metal contents were determined by the inductively coupled plasma spectroscopy (ICP-OES) model Varian 735, using a four-acid dissolution method for sample analysis (HF, HCl, HClO_4_, HNO_3_) [16]. The samples were weighed, and 0.250 g of HF (8ml) 40% and HClO_4_ (1ml) 70% was added. The solution was then relocated to a HOTBOX with water for max. 200°C until a gel solution was formed. Next, HCl (3.75 ml) 37% and HNO_3_ (1.25 M) 65% were decanted, and the solution was made to a volume of 25 ml, followed by the final analysis with ICP-OES. The quality assurance (QA) and quality control (QC) were evaluated by measuring blank and standard reference material NIST 2710. This provided an accuracy of 100 ± 5% (n = 15) whereas the precision of duplicate samples was 4–6%. To ensure the data quality of the samples, standard reference material (SRM) 2710 was simultaneously applied to 15% of the soil collected from the surface soil samples.

### Statistical analysis

Kolmogorov-Smirnov test (K-S) was employed to assess the normal distribution of data. Principal Component Analysis (PCA) and Cluster Analysis (CA) (Tokalioglu and Karatal, 2008) were also employed to identify the origins of heavy metal contaminations in surface soil samples. SPSS software package (version 20) was used for statistical analysis, and index calculations were performed using Excel software.

### Pollution assessment

The levels of soil contamination with heavy metals were evaluated and specified using different environmental indices:

#### Index of geoaccumulation (Igeo)

This index specifies the severity of the heavy metal pollution of soil and dust [17]. It is calculated as follows:

$$Igeo=Log_{2}(C_{n}/1.5*B_{n})$$ (1)

where Igeo is the index of geoaccumulation, C_n_ is the concentration of heavy metals measured in the sample, and B_n_ is the field content of the same heavy metal in the earth's crust. In this equation, a coefficient of 1.5 was used to correct the effects of the natural fluctuations of the materials on the environment. Based on this index, pollution can be classified into seven groups: no pollution (Igeo≥0), no to low pollution (0>Igeo≥1), low pollution (1>Igeo≥2), low to high pollution (2>Igeo≥3), high pollution (3>Igeo≥4), high to very high pollution (4>Igeo≥5), and very high pollution (Igeo<5) [18].

#### Enrichment factor (EF)

As a widespread formulation, enrichment factor (EF) is a simple method for assessing the extent of enrichment and comparing different environmental contaminants [19]. EF reflects the level of heavy metal pollution in the soil, and it is a useful index for distinguishing the natural and human sources of metals from each other [20]. In other words, EF is used to investigate the possible effects of human activities on the levels of heavy metals, which is calculated by Eq (2) [17].

$$EF=\frac{(\frac{CX}{Cref})Sample}{(\frac{Cx}{Cref})Background}$$ (2)

where $\left(\frac{CX}{Cref}\right)\;The\;sample,$ is the concentration ratio of the heavy metal measured in the soil to the field metal concentration in the sample; $\left(\frac{Cx}{Cref}\right)\;The\;background,$ is the concentration ratio of the sample metal to the base metal in the background values. Moroever, EF is the enrichment factor, C_x_ is the concentration of the element measured in the soil sample, and C_ref_ is the concentration of the reference element. In calculating EF, the reference element must have very little variability and be of a purely-geological origin. In certain environmental investigations, Zr, Ti, Fe, Al, and Sr have common uses as reference elements [21]. Therefore, the present research considered Al as a reference element as its human-related origins of contamination were not significant. The degree of heavy metal contamination was defined by the EF at five levels: low(EF> 2), moderate (2≥EF>5), high (5≥EF>20), very high (20≥EF>40), and extremely high (EF ≤40).

#### Potential ecological risk (PER)

Potential ecological risk (PER) assessment of heavy metals was first presented by Hakanson (1980). In the present study, the PER of heavy metals under analysis were determined by use of the following equations:

Cji=CiCji (3)

$$E_{j}^{i}=T_{n}^{i}\times C_{j}^{i}$$ (4)

$$RI=\sum_{i}^{n}E_{j}^{i}$$ (5)

In this relationship, C^i^ is the measured metal level in the soil sample, $C_{j}^{i}$ is the baseline reference value of that element, Tr is the toxicity response factor of each heavy metal (Cd, Cu, Pb, Cr, As, Zn, Vand Ni are 30, 5, 5, 2, 10, 1, 2 and 5) [22]. Furthermore, $E_{j}^{i}$ is the PER factor of each studied element, and RI is the PER of all elements. According to the ecological risk, the levels of heavy metal contamination were categorized into five levels, namely low PER (PER>40), moderate PER (40≥ PER> 80), significant PER (80 ≥ PER>160), high PER (160≥ PER >320), and very high PER (PER≤320). The contamination levels were classified into four categories based on the risk index: low (RI<150), moderate (150≥RI< 300), significant (300≥RI< 600), and high ecological risk (RI≥600).

#### Human health risk

The risk of carcinogenic and non-carcinogenic heavy metals was assessed (as a multi-stage process) in two parts based on the health risk assessment method proposed by the US Environmental Protection Agency (USEPA) [23]. In the assessment of both carcinogenic and non-carcinogenic risks, human exposure to metals was considered through ingesting, breathing in, and dermic exposure; also, the average daily dose value (ADD) in each route was calculated using Eqs 6–8 [24,25].

$$ADD_{ing}=\frac{C\times IngR\times CF\times EF\times ED}{BW\times AT}$$ (6)

$$ADD_{inh}=\frac{C\times InhR\times EF\times ED}{PEF\times BW\times AT}$$ (7)

$$ADD_{dermal}=\frac{C\times SA\times CF\times SL\times ABF\times EF\times ED}{BW\times AT}$$ (8)

where ADD_ing_, ADD_inh_, ADD_dermal_ are the average daily metal intake (mg/kg-day) by ingestion, inhalation, and dermal contact, respectively. C is the concentrations of metals in soil (mg/kg), IngR and InhR are the rates of soil ingestion and inhalation (mg/day and m^3^/day), EF is the exposure frequency to metals (day/year), CF is the conversion factor, ED is the exposure duration to metals (year), BW is the bodyweight of the person in contact with metals (Kg), AT is the averaging time (timespan during which exposure is averaged-days) to any amount of metals on a daily basis, SA is the skin area exposed to metals (cm^2^), SL is the skin adherence factor (mg/cm^2^), PEF is particle emission factor, and ABF is absorption factor (unitless). Table 1 depicts the details of each parameter and its values in the risk assessment. The average daily dose value of the metals (ADD) were calculated via ingestion, inhalation, and dermal contact; after that, thenon-cancer hazard quotient (HQ) was calculated based on the reference daily intake (*R*_*f*_*D*_*i*_) using Eq (9).

$${HQ}_{i}=\sum \frac{{ADD}_{i}}{R_{f}D_{i}}$$ (9)

**Table 1:** Exposure factor for metals doses.

Factor	Unit	Adult	Children	Reference
IngR	mg/day	100	200	[26]
InhR	m^3^/day	12.8	7.63	[26]
EF	day/year	350	350	[27]
ED	Year	24	6	[26]
BW	Kg	55.9	15	[28]
AT	Days	365×ED	365×ED	[29]
PEF	m^3^/kg	1.36E+09	1.36E+09	[26]
SA	cm^2^	4350	1600	[26]
AF	mg/cm^2^-day	0.7	0.2	[29]
ABF	-	0.001	0.100	[30]

HQ_i_ is a non-cancer hazard quotient in each intake path, ADD_i_ is the average daily dose value of metal intake by ingestion, inhalation, and dermal contact (mg/kg/day), and *R*_*f*_*D*_*i*_ is a reference of daily intake that estimatesthe maximal risk of heavy metals in the human population (adults and children) [31,32]. The *R*_*f*_*D*_*i*_ values of the studied metals were collected from the US Department of Energy's Risk Assessment Information System (RAIS) [27]. *R*_*f*_*D*_*i*_ at HQ ≤1 has no adverse effects on human health, but when HQ>1, a negative impact is expected. The total chronic noncancerous hazard index (HI) for adults and children can be generated in the three pathways to estimate the risk of all contaminated metals according to Eq (10).

$$HI=\sum {HQ}_{i}$$ (10)

The total chronic non-cancer hazard index (HI) calculated for all elements indicates the severity of undesirable effects in all pathways of human exposure. If HI ≤ 1, the total chronic non-cancer hazard is associated with a non-significant risk; an HI value of more than 1 implies that the total chronic non-cancer hazard is highly probable, which likelihood increases with an rise in the amount of HI [33]. The health risk assessment for carcinogenic heavy metal exposures for both adults and children was calculated through ingestion, inhalation, and dermal contact by Eq (11).

$$Carcinogenic\;risk(CR)=\sum {ADD}_{i}\times {SF}_{i}$$ (11)

In the above equation, CR is the cancer risk, and ADD_i_, SF_i_ is the risk factor for cancer per unit of exposure to metals (mg/kg/day). Generally, according to the US Environmental Protection Agency, negligible carcinogenic risk (CR) is less than 1×10^-6^ (the probability of one's cancer in every one million people) whereas if the CR is more than 1×10^-4^, it is hazardous to human health. CR ranging from 1×10^-6^ to 1×10^-4^ represents an acceptable risk under control and monitoring conditions [34]

## Results and discussion

### The measurement of heavy metal concentration

**Fig 2:**
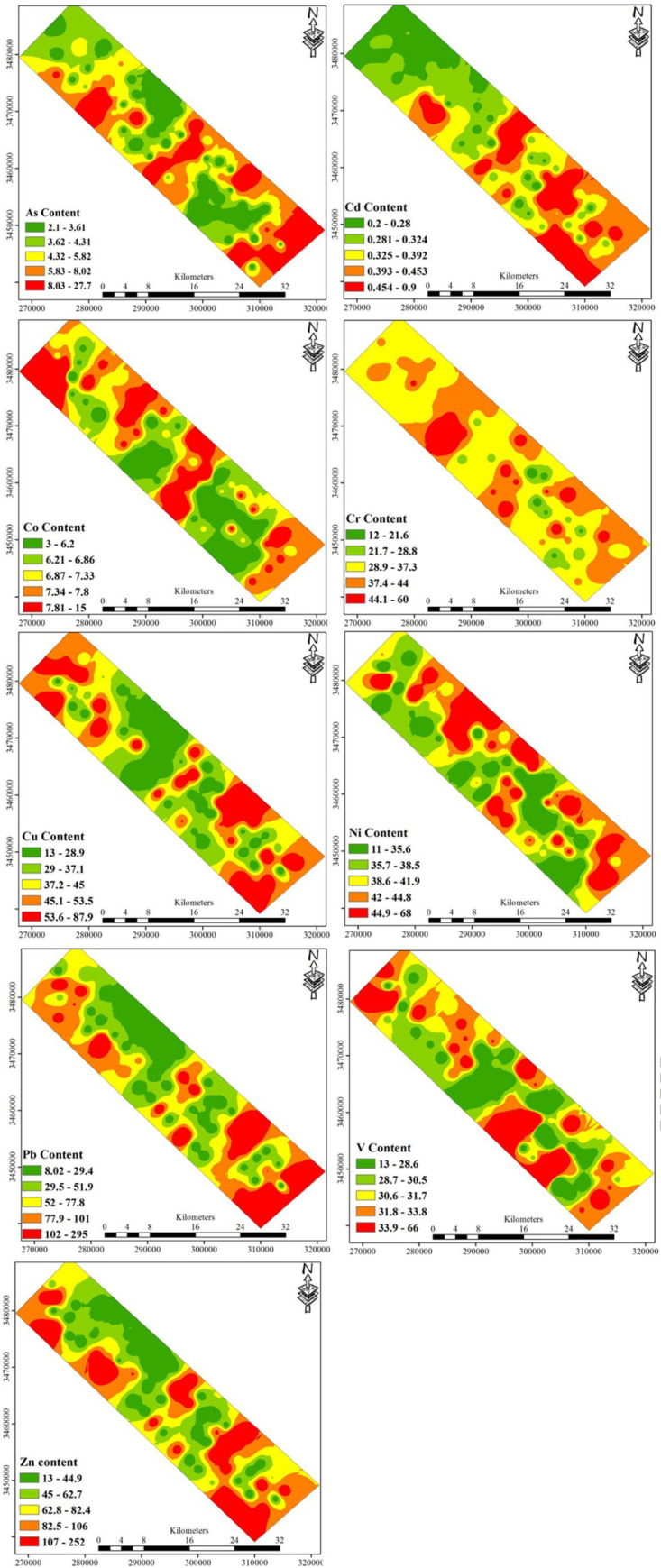
Spatial distribution of heavy metals in the surface soil of Ahvaz Oilfield.

Table 2 illustrates the statistical concentration data pertaining to the heavy metals in the soil of Ahvaz oil field. According to the results, the contents of As, Cd, Co, Cr, Cu, Ni, Pb, V, and Zn were 2-28, 0.2-0.9, 3-15, 12-60, 13-88, 11-68, 8-295, 13-66, and 13-252 (mg/kg), respectively. The average concentrations of these elements were 5.9, 0.4, 7.1, 36.5, 41.2, 39.8, 67.4, 31.5, and 77.6 mg/kg. The lowest and highest mean concentrations of heavy metals for Cd and Zn were obtained at 0.4 and 77.6 mg/kg. Concentrations of all studied elements, except for Co, Cr, and V, were higher than the baseline concentration (concentration in the earth’s crust). The high concentration level of these heavy metals is attributed to anthropogenetic activities(such as pollution) caused by oil refining and treatment activities in the region [35]. The main causes of heavy metal contamination in the studied oil-field are oil extraction and exploitation activities and combustion of petroleum hydrocarbon and gases in the flares operating in the area. Numerous studies have shown that Ni, Pb, and As are the cause of kidney and liver conditions and cancer problems in humans [36]. These metals, Pb in particular, can impact the environmental performance of areas contaminated with high concentrations [5]. Therefore, it is essential to reduce heavy metal concentration levels so as to prevent the potential hazards of metals to the environment and human health. The spatial distribution maps of heavy metals based on GIS methods can provide some information on the pollution sources of heavy metals in soils and their relative contribution from different sources. The spatial distribution of heavy metal concentrations can also be conducive to identifying their possible sources and certain pollution hot spots. Fig 2 illustrates the spatial distributions of Ni, Cr, Pb, Ni, Zn, and Cu contents, which were remarkably similar over a large area. Their high contents were mainly distributed in areas with a high density of oil well drilling activities, oil prodution, and oil de-saltation units; however, it was only in the middle parts of the study area that they were sparsely distributed (Fig 2).

**Table 2:** The summarized statistical parameters of heavy metal concentrations (mg/kg).

Element	Unit	Min-Max	Mean ± SD	Skewness	CV	Upper crust content
As	(mg/kg)	2-28	5.9 ± 6.1	2.4	1.1	2.1
Cd	(mg/kg)	0.2-0.9	0.4 ± 0.2	1.3	0.4	0.02
Co	(mg/kg)	3-15	7.1 ± 2	0.9	0.3	10
Cr	(mg/kg)	12-60	36.5 ± 10.2	-0.02	0.28	98
Cu	(mg/kg)	13-88	41.2 ± 24.1	0.6	0.6	33
Ni	(mg/kg)	11-68	39.8 ± 11.9	0.1	0.3	20
Pb	(mg/kg)	8-295	67.4 ± 70.5	1.2	1.1	15
V	(mg/kg)	13-66	31.5 ± 9.4	1.4	0.3	60
Zn	(mg/kg)	13-252	77.6 ± 62.8	1.1	0.8	28

### Identification of heavy metal sources

**Fig 3:**
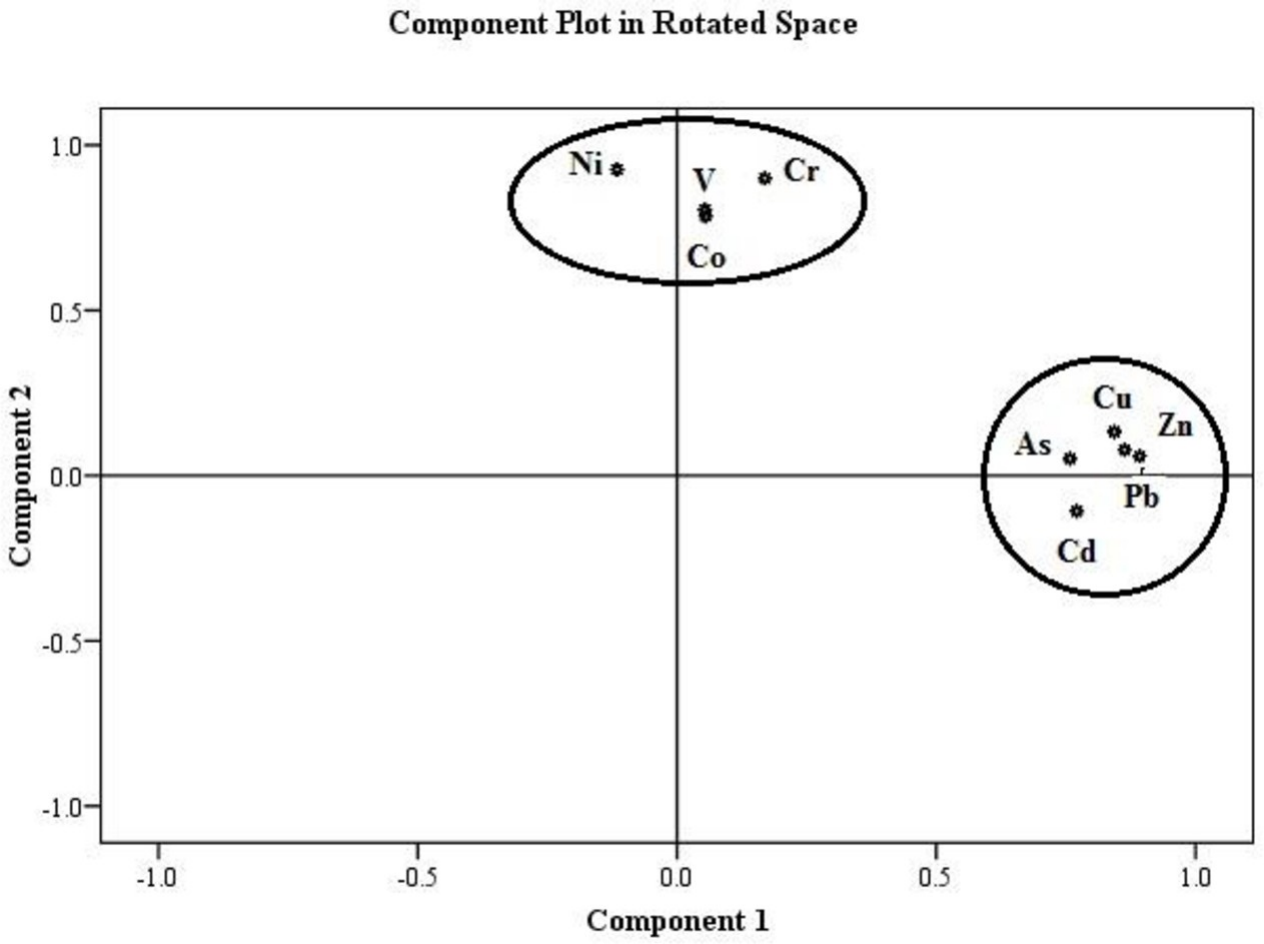
PCA of heavy metals in the surface soil of Ahvaz Oilfield.

**Fig 4:**
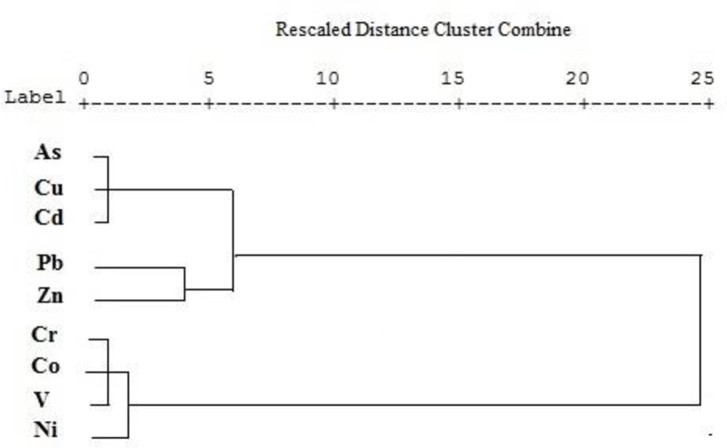
Cluster analysis (CA) of heavy metals in the surface soil of Ahvaz Oilfield.

Spearman's correlation coefficients were used to specify the associations between different studied heavy metals (Table 3). The correlation coefficient between the contaminants showed that at 0.01% level, Pb had a significant positive correlation with Zn (0.81), Cu (0.77), Cd (0.74), and As (0.70) because they had the same source of release into the environment. On the other hand, Cr, Ni, V, and Co were found to have a significant positive correlation at a 0.01% level, suggesting another cause of pollution for these elements. Principal Component Analysis (PCA) was utilized to examine the associations among heavy metals in the Ahvaz oil field and determine their potential sources of contamination. The PCA findings (Table 4) for heavy metal concentrations revealed that the two principal components (PCs) comprised 72% of the total variance. The first principal component (PC1) accounted for 39% of the total variance and included Pb, Zn, Cu, As, and Cd. The contamination source of these heavy metals is probably anthropogenic sources such as petroleum leakage [35] and the pollution caused by drilling mud from oil wells [2]. The latter was the second major component (PC2) accounting for 33% of the total variance and included Co, Cr, V, Ni. The present observations showed that the contamination source of heavy metals such as Co, V, and Cr was probably the natural sources because the concentrations of Co, Cr and V were lower than the baseline concentration (concentration in the earth’s crust); however, anthropogenic sources were the cause of Ni contamination in the study area (Fig 3). The results of CA and PCA were compared to ensure the accuracy of the results of the final identified sources. Accordingly, the examined heavy metals were divided into two main groups, the first one consisting of Pb, Zn, Cu, As, and Cd and the second one including Co, Cr, V, and Ni (Fig 4). These findings confirm the PCA results.

**Table 3:** Correlation among heavy metal concentrations in Ahvaz oil-field.

	As	Cd	Co	Cr	Cu	Ni	Pb	V	Zn
As	1.000								
Cd	0.566**	1.000							
Co	0.229	0.121	1.000						
Cr	0.433	0.156	0.774**	1.000					
Cu	0.729**	0.579**	0.248	0.291	1.000				
Ni	0.223	-0.021	0.761**	0.869**	0.076	1.000			
Pb	0.704**	0.734**	0.208	0.243	0.765**	0.012	1.000		
V	0.223	0.084	0.708**	0.811**	0.301	0.813**	0.248	1.000	
Zn	0.674**	0.778**	0.311	0.315	0.812**	0.095	0.805**	0.278	1.000

*Correlation is significant at 0.05 level (2-tailed).

**Correlation is significant at 0.01 level (2-tailed).

**Table 4:** Matrix of PCA loadings for toxic metal concentrations in the surface soils of Ahvaz oil field.

Element	Component		Communities
	1	2	
As	**0.703**	-0.285	0.576
Cd	**0.646**	-0.434	0.606
Co	0.400	**0.700**	0.650
Cr	0.546	**0.735**	0.837
Cu	**0.817**	-0.250	0.729
Ni	0.301	**0.883**	0.871
Pb	**0.810**	-0.308	0.751
V	0.393	**0.681**	0.618
Zn	0.828	-0.338	0.800
Initial Eigenvalue	3.470	2.970	
Total variance %	38.552	32.998	
Cumulative %	38.552	71550	

#### Index of geoaccumulation (Igeo)

The index of geo accumulation (Igeo) is commonly employed to evaluate regional environmental quality [11]. Table 5 presents the Igeo accumulation values of heavy metals. The results showed that the average Igeo of the studied heavy metals in soil samples decreased as follows: Pb > As > Zn > Ni > Cu > >Cd > Co > V > Cr. The contamination level of each element in the study area was based on the average Igeo: V, Cu, Cr, and Co were non-contaminated (Igeo≥ 0) while As, Cd, Ni, Zn, and Pb were of non to slightly contaminated levels (0>PI≥1). Additionally, according to the results shown in Table 5, V and Cr with 66 samples (100%) and Co with 65 samples (98.49%) were non-contaminated. On the other hand, Ni with 52 samples (78.79%), As with 33 samples (50%), and Zn with 32 samples (48.48%) had low to very high levels of contamination. With 31 samples (43.94%), Pb was slightly to severely contaminated.

**Table 5:** Statistical results of the Igeo of heavy metals in the study area.

Igeo Number of samples (%)								
Metals	Min	Max	Mean	Non polluted	Non polluted to slightly polluted	Slightly to very polluted	very polluted	Very to extremely polluted
As	-0.58	3.14	0.47	33(50%)	18(27.27%)	8(9.09%)	6(12.49%)	1(1.15%)
Cd	-0.58	1.58	0.24	38(57.58%)	17(25.75%)	11(16.67%)	**-**	**-**
Co	-2.32	0.01	-1.14	65(98.49%)	1(1.15%)	-	**-**	**-**
Cr	-3.61	-1.29	-2.07	66(100%)	-	-	**-**	**-**
Cu	-1.93	0.83	-0.52	42(63.63%)	24(36.37%)	-	**-**	**-**
Ni	-1.45	1.18	0.34	14(21.21%)	49(74.24%)	3(4.55%)	**-**	**-**
Pb	-1.67	3.53	0.49	35(53.03%)	2(3.03%)	10(15.15%)	15(22.72%)	4(6.07%)
V	-2.79	-0.45	-1.57	66(100%)	-	-	-	-
Zn	-1.69	2.58	0.42	34(51.52%)	9(13.63%)	15(22.73%)	8(12.12%)	-

### Enrichment factor (EF)

The enrichment factor (EF) was further investigated to specify the anthropogenic or natural sources of heavy metals in the surface soil of the Ahvaz oil field. Table 6 shows the enrichment factor (EF) values of heavy metals. As observed, the mean EF of the studied heavy metals in the sampled soil was reduced as Pb > Zn > As > Cd > Cu > Co > V > Ni > Cr. Based on mean EF, the contamination level of each element in the research zone was as follows: V, Ni, and Cr caused moderate enrichment (2≥EF<5), Co, Cu, Cd, and As were of high enrichment level (5≥EF<20), and Zn and Pb had very high enrichment (20≥EF<40). Furthermore, according to the results shown in Table 5, V with 53 samples (80.3%), Ni with 58 samples (88%), and Cr with 58 samples (88%) were of moderate enrichment levels. Furthermore, Cd with 51 samples (77.3%), Co with 50 samples (75.8%), Cu with 42 samples (63.6%), As with 34 samples (51.5%), and Zn with 38 samples (57.8%) had high enrichment levels. Meanwhile, with 30 samples (45.4%), Pb had a very high enrichment level. The present observations revealed that the values of pollution index varied among the studied heavy metals.Heavy metal pollution levels, on the other hand, indicated the severe contamination caused by heavy metals [20,37] and their anthropogenic sources in the soil of the study area [22].

**Table 6:** Statistical results of the heavy metal enrichment factor (EF) in the study area.

Enrichment factor (EF)				Number of samples (%)				
Metals	Min	Max	Mean	Low enrichment	Moderate enrichment	High enrichment	Very high enrichment	Extremely high enrichment
As	7.9	51.2	17.3	-	10(15.2%)	34(51.5%)	15(22.7%)	7(10.6%)
Cd	7.8	48.9	16.4	-	-	51(77.3)	13(19.7%)	2(3%)
Co	3.3	13.2	5.9	-	16(24.2%)	50(75.8%)	-	-
Cr	1.1	5.7	3.1	4(6%)	58(88%)	4(6%)	-	-
Cu	3.4	26.9	10.6	-	16(24.2%)	42(63.6%)	8(12.2%)	-
Ni	0.9	6.1	3.4	5(7.6%)	58(87.9%)	3(4.5%)	-	-
Pb	3.9	133.5	33.7	-	5(7.6%)	31(47%)	4(6%)	26(39.4%)
V	1.8	11.7	4.4	1(1.5%)	53(80.3%)	12(18.2%)	-	-
Zn	4.9	94.3	24	-	1(1.5%)	38(58.7%)	15(22.7%)	12(18.2%)

### Potential ecological risk (PER)

The potential ecological risk of heavy metals in the study area was measured based on the Hakanson method [38]. The values of PER and RI (Tables 7 and 8) indicate that the mean PER for the studied heavy metals in soil samples was reduced as Cd > As > Pb > Ni > Cu > >Co > Zn > V > Cr. Based on the average amounts of PER for heavy metals, Cr, V, Zn, Co, Cu, Ni, Pb, and As had low PER (PER> 40) whereas Cd had a moderate PER (40≥PER>80). Based on the results, the RI of all samples varied from at least 52 to a maximum of 311. Based on the mean RI (130), the samples were of low risk (RI>150). It was further observed that 44 samples (66.66%) had low risk, 20 samples (30.30%) had moderate risk, and two samples (3.04%) were of significant risk. Cd was identified as an effective factor in increasing PER in Ahvaz oil field; the prolonged exposure to this element in the surface soil can entail severe toxicity in humans and lead to kidney diseases. Cd in the surface soil candamage the internal tissues of children and adults through dermal contact [36]. It can further impact the ecological performance of areas with high concentrations [5].

**Table 7:** Statistical results related to the PER factor of heavy metals in the study area.

PER factor					
Skewness	SD	Min	Max	Mean	Metals
2.4	28.8	10	131.9	28.1	As
1.3	25.4	30	135	57.4	Cd
0.9	1	1.5	7.5	3.5	Co
-0.1	0.2	0.2	1.2	0.8	Cr
0.6	3.7	2	13.3	6.2	Cu
0.1	3	2.8	17	9.9	Ni
1.2	20.7	2.4	86.8	19.8	Pb
1.4	0.3	0.4	2.2	1.1	V
1	2.2	0.5	9	2.8	Zn

**Table 8:** Statistical results of Risk Index (RI) of heavy metals in the study area.

RI			Number of samples			
Mean	Max	Min	Low risk	Moderate risk	Significant risk	High risk
130	311	52	44(66.66%)	20(30.30%)	2(3.04)	-

### Human health risk

We assessed the human health risk in the soil samples of Ahvaz oil field in contact with metals via the three main pathways of ingestion, inhalation, and dermal contact in children and adults. Table 9 shows the hazard quotient (HQ) and the hazard index (HI) of all the three pathways regarding each heavy metal. The highest and lowest levels of HQ in both age groups were as follows: ingestion > dermal contact > inhalation, respectively. Moreover, the HQ levels in ingestion and inhalation pathways were higher in children than in adults; the HQ levels in the dermal contact pathway, on the other hand, were higher in adults than in children. Also, in both age groups, the highest HQ belonged to to As taken through ingestion. Overall, the HQ of heavy metals in all three pathways was less than 1. Thus, they would have no adverse effects on humans, as was confirmed by the results of Liu et al. [39] and Wei et al. [34]. Tao et al. [40] reported that in children, the risk of exposure to heavy metals during ingestion was higher than that of inhalation and dermal contact. Similarly, according to Table 9, the hazard index (HI) values of all three pathways for children were 2.14-6.96 folds higher than those for adults. In addition, the HI of all heavy metals in the soil of Ahvaz oil field were 0.72 and 0.14 for children and adults, respectively. This implies that children are more exposed to the risk of heavy metals in comparison to adults.The order of HI was As > Pb > Cr > Ni > Cu > Cd > Zn for children and Cr > Pb > As > Ni > Cd > Cu > Zn for adults. The HI values of heavy metals were less than 1, showing that the HI is low for heavy metals [23]. The HI values were much higher in children than in adults, which is consistent with our results. Table 9 shows the cancer risk (CR) assessment of heavy metals for children and adults, which follows Cr > Ni > As > Pb > Cd. Therefore, in both age groups, Cr had the highest CR while Cd had the least CR. In children, CR values were higher than those in adults. The CR of As, Ni, Cd, and Pb in children and adults was less than 1×10^-6^, indicating that the CR of these metals in surface soil can be neglected. On the contrary, in children and adults, the CR of Cr was greater than 1×10^-6^. This indicates that the cancer risk of Cr requires immediate attention particularly because children are more susceptible to human health risks of heavy metals. Chabukdhara and Nema [30] also reported that in the industrial zone of India, the highest CR after Cr belonged to Ni, Pb, and Cd. Our findings revealed that CR was higher in children than in adults, which is in accordance with the results of Qing et al. [23].

**Table 9:** Health risk factors from heavy metal in the soil of Ahvaz oil field.

(mg/kg)	Pb	Zn	Cu	Cr	Cd	Ni	As
C(95% UCL)	67.42	77.61	41.17	36.52	0.38	39.83	5.89
RfD_ing_ (mg/kg day)	3.00E - 03	3.00E - 01	4.00E - 02	3.00E - 03	1.00E - 03	2.00E-02	3.00E - 04
RfD_inh_ (mg/kg day)	3.52E - 03	3.00E - 01	4.02E - 02	2.86E - 05	1.00E - 03	2.02E-02	3.10E - 04
RfD_derm_ (mg/kg day)	5.25E - 04	6.00E - 02	1.20E - 02	6.00E - 05	1.00E - 05	5.40E-03	1.23E - 04
SF (mg/kg day)^-1^	-	-	-	4.20E + 01	6.30E + 00	8.40E-01	1.51E +01
Children							
HQ_ing_	2.46E-01	3.33E-03	131E-02	1.56E-01	4.85E-03	2.54E-02	2.51E-01
HQ_inh_	6.86E-06	9.27E-08	3.67E-07	4.58E-04	1.36E-07	6.93E-07	6.81E-06
HQ_derm_	2.62E-03	2.64E-05	7.01E-05	1.25E-02	7.78E-04	1.51E-04	9.79E-04
HI = ∑HQi	2.48E-01	3.33E-03	1.32E-02	1.69E-01	5.64E-03	2.56E-02	2.52E-01
CR	2.66E-08	-	-	1.44E-05	2.25E-08	3.15E-07	8.31E-08
Adults							
HQ_ing_	3.30E-02	4.43E-04	1.76E-03	2.09E-02	6.51E-04	3.41E-03	3.37E-02
HQ_inh_	4.81E-06	6.52E-08	2.58E-07	3.22E-04	9.58E-08	4.87E-07	4.79E-06
HQ_derm_	6.70E-03	6.75E-05	1.79E-04	3.17E-02	1.98E-03	3.85E-04	2.51E-03
HI = ∑HQi	3.98E-02	5.11E-04	1.94E-03	5.30E-02	2.63E-03	3.81E-03	3.62E-02
CR	3.57E-09	-	-	1.93E-06	3.02E-09	4.22E-08	1.11E-08

## Conclusion

We determined the concentration values, index of geoaccumulation, enrichment factors, and ecological and human health risk of potentially heavy metals (Pb, Zn, Cu, Cd, Cr, As, Ni, Co, and V) in the surface soil of Ahvaz oil field; all heavy metals, with the exception of Co, Cr, and V in the surface soil were higher than their corresponding upper crust content (UCC), hence potential anthropogenic sources. Based on the correlation coefficients, cluster analysis (CA), and principal component analysis (PCA), major sources of heavy metals in the soils of Ahvaz oil field were mainly the petroleum exploration and production operations as well as the combustion of petroleum gases and hydrocarbons in the flares operating in Ahvaz oil field and their deposition in the soil of the area. The Igeo values in the surface soil decreased in the following order: Pb > As > Zn > Ni > Cu > >Cd > Co > V > Cr. As, Cd, Ni, Zn, and Pb were of non to slightly contaminated levels. The EF values in the surface soil were reduced as follows: Pb > Zn > As > Cd > Cu > Co > V > Ni > Cr. Lead and Zn were of very high EF mean enrichment values, and Co, Cu, Cd, and As had high enrichment in the surface soil, suggesting that the soil in this study area is significantly influenced by human activities. Pb, Cr, V, Zn, Co, Cu, Ni, and As had a low PER while Cd had a moderate PER. The findings also revealed that all samples had a low risk based on the mean RI. Human health risk assessment of the studied metals in the surface soil samples indicated the following order of exposure pathways for the studied potentially heavy metals: ingestion > dermal contact > inhalation. The hazard quotient (HQ) of potentially heavy metals in the surface soil samples of Ahvaz oil field was higher in children than in adults. The HI value related to each metal was < 1 (close enough), which suggests no non-carcinogenic risk for both target populations. The risk of carcinogenic and non-carcinogenic diseases was detected to be higher in children than in adults. The Cr carcinogenic risk calculation was more than 1 × 10^-6^ for children and adults. Ultimately, the CR of Cr for both children and adults indicated risk under control conditions.

## Supporting information

S1 Data (XLSX)

## References

[1] Ghanavati N, Nazarpour A, Watts MJ. Status, source, ecological and health risk assessment of toxic metals and polycyclic aromatic hydrocarbons (PAHs) in street dust of Abadan, Iran. Catena. 2019;177:246–59.

[2] Babaei H, Ghanavati N, Nazarpour A. Contamination level of mercury in the street dust of ahvaz city and its spatial distribution. JWSS-Isfahan University of Technology. 2018;22(3):249–59.

[3] Alloway BJ. Sources of heavy metals and metalloids in soils Heavy metals in soils: Springer; 2013 p. 11–50.

[4] Nazarpour A, Ghanavati N, Babaenejad T. Evaluation of the level of pollution and potential ecological risk of some heavy metals in surface soils in the Ahvaz oil-field. Iranian Journal of Health and Environment. 2017;10(3):391–400.

[5] Ogunkunle CO, Fatoba PO. Pollution Loads and the Ecological Risk Assessment of Soil Heavy Metals around a Mega Cement Factory in Southwest Nigeria. Polish Journal of Environmental Studies. 2013;22(2).

[6] Nazarpour A, Watts MJ, Madhani A, Elahi S. Source, spatial distribution and pollution assessment of Pb, Zn, Cu, and Pb, isotopes in urban soils of Ahvaz City, a semi-arid metropolis in southwest Iran. Scientific reports. 2019;9(1):1–11. 10.1038/s41598-018-37186-2 30926876 PMC6441049

[7] Fatoba PO, Ogunkunle CO, Ihaza CO. Assessment of metal pollution of soil and Diagnostic species associated with oil spills in the Niger Delta, Nigeria. Environmental Research, Engineering and Management. 2015;71(3):13–22.

[8] Ite AE, Ibok UJ, Ite MU, Petters SW. Petroleum exploration and production: Past and present environmental issues in the Nigeria’s Niger Delta. American Journal of Environmental Protection. 2013;1(4):78–90.

[9] Adesina GO, Adelasoye KA. Effect of crude oil pollution on heavy metal contents, microbial population in soil, and maize and cowpea growth. Agricultural sciences. 2014;2014.

[10] Chen H, Teng Y, Lu S, Wang Y, Wang J. Contamination features and health risk of soil heavy metals in China. Science of the total environment. 2015;512:143–53. 10.1016/j.scitotenv.2015.01.025 25617996

[11] Cheng J-l, Zhou S, Zhu Y-w. Assessment and mapping of environmental quality in agricultural soils of Zhejiang Province, China. Journal of Environmental Sciences. 2007;19(1):50–4. 10.1016/s1001-0742(07)60008-4 17913153

[12] Keshavarzi B, Tazarvi Z, Rajabzadeh MA, Najmeddin A. Chemical speciation, human health risk assessment and pollution level of selected heavy metals in urban street dust of Shiraz, Iran. Atmospheric Environment. 2015;119:1–10.

[13] Ebrahimi S, Shayegan J., Malakouti M.J. and Akbari A. Environmental Evaluation and Assessment of Some Important Factors of Oil Contamination in Soil around Sarkhoun Gas Refinery of Bandar Abbas. Journal of Environmental studies. 2011;57:9–26.

[14] Fasihi H, Hamidi M, Ostadfarag S. Investigation of heavy metals and hydrocarbons contamination in Baghershar, Tehran, IRAN. 2017;10:19–32.

[15] Borojerdnia A, Rozbahani MM, Nazarpour A, Ghanavati N, Payandeh K. Application of exploratory and Spatial Data Analysis (SDA), singularity matrix analysis, and fractal models to delineate background of potentially toxic elements: A case study of Ahvaz, SW Iran. Science of The Total Environment. 2020:140103. 10.1016/j.scitotenv.2020.140103 32559546

[16] Yuen J, Olin PH, Lim H, Benner SG, Sutherland R, Ziegler A. Accumulation of potentially toxic elements in road deposited sediments in residential and light industrial neighborhoods of Singapore. Journal of Environmental Management. 2012;101:151–63. 10.1016/j.jenvman.2011.11.017 22410188

[17] Wei B, Yang L. A review of heavy metal contaminations in urban soils, urban road dusts and agricultural soils from China. Microchemical journal. 2010;94(2):99–107.

[18] Gonzalez-Macias C, Schifter I, Lluch-Cota D, Mendez-Rodriguez L, Hernandez-Vazquez S. Distribution, enrichment and accumulation of heavy metals in coastal sediments of Salina Cruz Bay, Mexico. Environmental monitoring and assessment. 2006;118(1-3):211–30. 10.1007/s10661-006-1492-8 16897543

[19] Addo M, Darko E, Gordon C, Nyarko B, Gbadago J. Heavy metal concentrations in road deposited dust at Ketu-south district, Ghana. 2012.

[20] Yang Z, Lu W, Long Y, Bao X, Yang Q. Assessment of heavy metals contamination in urban topsoil from Changchun City, China. Journal of Geochemical Exploration. 2011;108(1):27–38.

[21] Abrahim G, Parker R. Assessment of heavy metal enrichment factors and the degree of contamination in marine sediments from Tamaki Estuary, Auckland, New Zealand. Environmental monitoring and assessment. 2008;136(1-3):227–38. 10.1007/s10661-007-9678-2 17370131

[22] Lu X, Wang L, Li LY, Lei K, Huang L, Kang D. Multivariate statistical analysis of heavy metals in street dust of Baoji, NW China. Journal of hazardous materials. 2010;173(1-3):744–9. 10.1016/j.jhazmat.2009.09.001 19811870

[23] Qing X, Yutong Z, Shenggao L. Assessment of heavy metal pollution and human health risk in urban soils of steel industrial city (Anshan), Liaoning, Northeast China. Ecotoxicology and environmental safety. 2015;120:377–85. 10.1016/j.ecoenv.2015.06.019 26114257

[24] Du Y, Gao B, Zhou H, Ju X, Hao H, Yin S. Health risk assessment of heavy metals in road dusts in urban parks of Beijing, China. Procedia Environmental Sciences. 2013;18:299–309.

[25] Kurtz JC, Jackson LE, Fisher WS. Strategies for evaluating indicators based on guidelines from the Environmental Protection Agency’s Office of Research and Development. Ecological indicators. 2001;1(1):49–60.

[26] USEPA. Baseline Human Health Risk Assessment Vasquez Boulevard and I-70 Superfund Site, Denver, CO. 2001.

[27] Ferreira-Baptista L, De Miguel E. Geochemistry and risk assessment of street dust in Luanda, Angola: a tropical urban environment. Atmospheric environment. 2005;39(25):4501–12.

[28] Shi G, Chen Z, Bi C, Wang L, Teng J, Li Y, et al A comparative study of health risk of potentially toxic metals in urban and suburban road dust in the most populated city of China. Atmospheric Environment. 2011;45(3):764–71.

[29] USEPA I. Reference Dose (RfD): Description and Use in Health Risk Assessments, Background Document 1A, Integrated Risk Information System (IRIS). USEPA Washington DC; 1993.

[30] Chabukdhara M, Nema AK. Heavy metals assessment in urban soil around industrial clusters in Ghaziabad, India: probabilistic health risk approach. Ecotoxicology and environmental safety. 2013;87:57–64. 10.1016/j.ecoenv.2012.08.032 23116622

[31] Man YB, Sun XL, Zhao YG, Lopez BN, Chung SS, Wu SC, et al Health risk assessment of abandoned agricultural soils based on heavy metal contents in Hong Kong, the world's most populated city. Environment international. 2010;36(6):570–6. 10.1016/j.envint.2010.04.014 20552725

[32] Thompson KM, Burmaster DE, Crouch EA3. Monte Carlo techniques for quantitative uncertainty analysis in public health risk assessments. Risk Analysis. 1992;12(1):53–63. 10.1111/j.1539-6924.1992.tb01307.x 1574617

[33] Assessment PR. Risk Assessment Guidance for Superfund: Volume III-Part A. 2001.

[34] Wei X, Gao B, Wang P, Zhou H, Lu J. Pollution characteristics and health risk assessment of heavy metals in street dusts from different functional areas in Beijing, China. Ecotoxicology and environmental safety. 2015;112:186–92. 10.1016/j.ecoenv.2014.11.005 25463870

[35] Fu X, Cui Z, Zang G. Migration, speciation and distribution of heavy metals in an oil-polluted soil affected by crude oil extraction processes. Environmental Science: Processes & Impacts. 2014;16(7):1737–44. 24824116 10.1039/c3em00618b

[36] Bada BS, Olarinre TA, editors. Characteristics of soils and heavy metal content of vegetation in oil spill impacted land in Nigeria. proceedings of the Annual International Conference on Soils, sediments, Water and Energy; 2012.

[37] Mmolawa K, Likuku A, Gaboutloeloe G. Assessment of heavy metal pollution in soils along major roadside areas in Botswana. African Journal of Environmental Science and Technology. 2011;5(3):186–96.

[38] Hakanson L. An ecological risk index for aquatic pollution control. A sedimentological approach. Water research. 1980;14(8):975–1001.

[39] Liu X, Song Q, Tang Y, Li W, Xu J, Wu J, et al Human health risk assessment of heavy metals in soil–vegetable system: a multi-medium analysis. Science of the Total Environment. 2013;463:530–40. 10.1016/j.scitotenv.2013.06.064 23831799

[40] Tao X-Q, Shen D-S, Shentu J-L, Long Y-Y, Feng Y-J, Shen C-C. Bioaccessibility and health risk of heavy metals in ash from the incineration of different e-waste residues. Environmental Science and Pollution Research. 2015;22(5):3558–69. 10.1007/s11356-014-3562-8 25249049

